# Low expression of SEMA6C accelerates the primordial follicle activation in the neonatal mouse ovary

**DOI:** 10.1111/jcmm.13337

**Published:** 2017-09-07

**Authors:** Su Zhou, Wei Yan, Wei Shen, Jing Cheng, Yueyue Xi, Suzhen Yuan, Fangfang Fu, Ting Ding, Aiyue Luo, Shixuan Wang

**Affiliations:** ^1^ Department of Obstetrics and Gynecology Tongji Hospital Tongji Medical College Huazhong University of Science and Technology Wuhan Hubei China

**Keywords:** *Sema6c*, primordial follicle activation, PI3K‐AKT‐rpS6, ovarian reserve

## Abstract

The primordial follicle assembly, activation and the subsequent development are critical processes for female reproduction. A limited number of primordial follicles are activated to enter the growing follicle pool each wave, and the primordial follicle pool progressively diminishes over a woman's life‐time. The number of remaining primordial follicles represents the ovarian reserve. Identification and functional investigation of the factors involved in follicular initial recruitment will be of great significance to the understanding of the female reproduction process and ovarian ageing. In this study, we aimed to study whether and how semaphorin 6C (*Sema6c*) regulated the primordial follicle activation in the neonatal mouse ovary. The attenuation of SEMA6C expression by SiRNA accelerated the primordial follicle activation in the *in vitro* ovary culture system. PI3K‐AKT‐rpS6 pathway was activated when SEMA6C expression was down‐regulated. And the LY294002 could reverse the effect of low SEMA6C expression on primordial follicle activation. Our findings revealed that *Sema6c* was involved in the activation of primordial follicles, and the down‐regulation of SEMA6C led to massive primordial follicle activation by interacting with the PI3K‐AKT‐rpS6 pathway, which might also provide valuable information for understanding premature ovarian failure and ovarian ageing.

## Introduction

Once the primordial follicles are activated, these follicles are destined either to undergo atresia or to ovulate. Under physiological conditions, every cycle only a limited number of primordial follicles are activated to step into the growing follicle pool 1. The female reproductive capability and ovarian lifespan are determined by the regulation of primordial follicle initial recruitment 2. With the female ageing, the primordial follicle number declines, the size of small antral follicles decreases, and the oocyte quality deteriorates. The factors that regulate the activation of the primordial follicle are of great importance for female fertility as well as for ovarian ageing.

Under physiological conditions, some primordial follicles remain dormant, while others enter the growth phase. The activation of primordial follicles is a process of precise regulation. Numerous growth factors, cytokines and bidirectional signalling between oocytes and granulosa cells are important and necessary to this process 3, 4, 5, 6. Recent studies using genetically modified mouse models have revealed that the PTEN/PI3K/AKT/FOXO3A and TSC/mTOR1 signalling pathways are important for the regulation of primordial follicle activation 7, 8, 9, 10, 11, 12, 13. Besides, another recent study demonstrated that *Lhx8* regulates postnatal folliculogenesis and primordial follicle activation through interaction with PI3K‐AKT pathway *via* regulating *Lin28a* expression. Conditional depletion of *Lhx8* by *Gdf9‐Cre* could lead to massive primordial follicle activation 14. A recent study also revealed the role of LKB1 as an indispensable gatekeeper for the primordial follicle pool through elevated mTOR signalling by an AKT‐independent LKB1‐AMPK pathway 15. Expression change of anyone of the above‐mentioned key genes may lead to different fate of the primordial follicle pool and reproductive productivity. Excessive activation of primordial follicles or cell death may lead to premature ovarian failure. However, the mechanisms by which some primordial follicles are activated, while others stay in a quiescent state are little known up to now.

Semaphorins were initially described in the nervous system based on their role in repulsive axon guidance, but now they act as crucial regulators of homeostasis and morphogenesis on more organ systems 16, 17. The structures of semaphorins are well conserved in rodents and human. The diverse characteristics of semaphorin signalling and its implication in disease have attracted great attention on the study of biological effects of semaphorins 16, 17, 18. Nowadays, more studies about semaphorins are performed in cancer, immune system, vascular system and organogenesis *et al*. 17, 19, 20, 21, 22. Several studies have demonstrated that semaphorin family participates in the regulation of follicle development. *Sema‐4D/Plexin‐B1* system could influence the follicular growth as well as the steroidogenesis 23. Semaphorins (*Sema3a, Sema6a and Sema6d*), as cell‐to‐cell communication genes, play an important role in the maturation of the oocyte cumulus complex 24. SEMA3A is up‐regulated in the GnRHa‐triggered follicle compared with that in the hCG‐triggered follicle, which suggests an impaired induction of angiogenesis in the GnRHα‐triggered MGC (mural granulosa cells) as compared with the hCG‐triggered MGC 25. SEMA6C, a member of the semaphorin family, has been demonstrated to interact with PLEXIN A1, which can regulate the axon repulsion and has growth cone collapsing activity 21, 22. This study aimed to explore the role of *Sema6c* in the process of primordial follicle activation and its signalling regulation in the neonatal mouse ovary.

## Materials and methods

### Animals

Healthy C57BL/6J female mice were purchased from the Center for Laboratory Animal Administration of the Center for Disease Control and Prevention of Hubei Province (Wuhan, China). They were bred with free access to food and water under a light/dark cycle (12 hrs/12 hrs) at the Center for Laboratory Animal Administration of Tongji Medical College, Huazhong University of Science and Technology (Wuhan). The 6‐ to 8‐week‐old female mice were mated with adult male mice in specific pathogen‐free conditions. The 3‐day‐old neonatal mouse ovaries were used for the experiment. All these mice received humane care according to the Guide for the Care and Use of Laboratory Animals of the Chinese Academy of Sciences.

### Immunohistochemistry

The ovaries of the neonatal mice (Day 3, Day 5 and Day 7) were fixed in 4% paraformaldehyde for 24 hrs, transferred to a graded series of ethanol to dehydrate, embedded in paraffin and serially sectioned (5 μm). The ovary sections were processed for immunohistochemical analysis using routine immunohistochemistry procedures. The antigen was retrieved by citric acid buffer (PH 6.0) microwave antigen retrieval method. The citric acid buffer was heated to boiling in a microwave oven (M1‐L213B; Midea, Guangdong, China); tissue sections after dewaxing and hydration were placed into the boiling buffer; and then, the sections were heated in the microwave oven as following: medium fire (600 Watts) 8 min., ceasefire 8 min., low fire (500 Watts) 7 min. The ovary sections were incubated with anti‐SEMA6C primary antibody (AF2108, 1:100, 2 μg/ml; R&D Systems, Minneapolis, MN, USA) overnight at 4°C. The next day, the ovary sections were incubated with a secondary antibody (HRP‐labelled rabbit Anti‐goat IgG (H + L), Servicebio, GB23204, 1:200) for 50 min. at 37°C. Subsequently the ovary sections were visualized with DAB‐HRP chromogenic agent (K5007; DAKO, Glostrup, DK) at room temperature for 20 sec. As the negative control, normal goat IgG (BA1044, 2 μg/ml; BOSTER, Wuhan, China) instead of the primary antibodies was used to treat the sections. Microscopy was performed using microscope (version 1.8.1; Olympus, Japan), and the images were acquired with the cellSens Dimension software (version 1.8.1, Olympus, Japan).

### *In vitro* ovary culture

On postnatal Day 3, the female mice were killed by decapitation. Ovaries were removed, and then, the oviduct and excess tissue were trimmed under the stereomicroscope. The ovaries were placed on a piece of Millicell‐CM filter membrane (Millipore, Billerica, MA, USA) floating in 400 μl of alpha‐modified Eagle's medium (Invitrogen, Carlsbad, CA, USA) containing 1 mg/ml Albumax (Invitrogen), 1 mg/ml BSA (Sigma‐Aldrich, St. Louis, MO, USA), 50 μg/ml L‐ascorbic acid (Sigma‐Aldrich), 0.23 mmol/l Na pyruvate (Sigma‐Aldrich), ITS (1.0 mg/ml insulin, 0.55 mg/ml human transferrin and 0.5 μg/ml sodium selenite) (Sigma‐Aldrich), 5 U/ml penicillin and 5 μg/ml streptomycin, as the study described before 26. The culture medium was changed every other day together with the intervention factor.

### SiRNA transfection

Cultured ovaries were treated with 2 μM/4 μM cholesterol‐conjugated negative control SiRNA (NC‐SiRNA) (Ribobio, China, NControl_05815, siN05815122147) or cholesterol‐conjugated Si‐*Sema6c* RNA (Si‐*Sema6c*‐Chol) (Ribobio, China, siG0931383544) for 48 hrs to determine the optimal intervention concentration. Follow‐up experiments were conducted with the best intervention concentration for 4 days. The SiRNA sequences used for silencing *Sema6c* (Gene ID: 20360) were designed and synthesized by Guangzhou Ribobio company (Ribobio, China). The sequences of Si‐*Sema6c*‐RNAs were as follows: 5′ CCAGUGAUGCUGUAGUUUA dTdT 3′, 3′ dTdT GGUCACUACGACAUCAAAU 5′. The SiRNAs were cholesterol‐conjugated and Cy3‐labelled to better enter the cells and be observed conveniently. The cholesterol‐conjugated SiRNA has been demonstrated effective to down‐regulate the gene expression. The PI3K‐specific inhibitor LY294002 purchased from Selleckchem (S1105; Selleckchem, Houston, TX, USA) was added to the 48 hrs transfected ovary for another 48 hrs. The concentration of LY294002 added to the medium was 20 μM.

### Fluorescence microscopy

To determine whether the Si‐*Sema6c*‐Chol could enter the ovary, the transfected ovaries were placed under the fluorescence microscope to observe the CY3 fluorescence after 48‐hrs transfection. Images were photographed using the program cellSens Dimension by fluorescence microscope (version 1.8.1, Olympus, Japan).

### Histology and immunofluorescence analysis

Ovaries, following 4 days of *in vitro* culture, were fixed in 4% paraformaldehyde and then dehydrated, embedded in paraffin and serially sectioned 5 μm per section. Two of the largest cross sections were stained with haematoxylin and eosin (HE). The MVH (mouse vasa homolog) is specifically expressed in the germ cell lineage 27, 28 and could be used as a germ cell‐specific marker to label the oocytes of all stage follicles in the mouse ovaries 29. Zona pellucida glycoprotein 3 (ZP3) is expressed specific in the growing follicles when the primordial follicles are activated 30. Therefore, ZP3 antibody (H‐300) (sc‐25802; Santa Cruz Biotechnology, Santa Cruz, CA, USA) was also used for immunofluorescence to count growing follicles more accurately. The sections right adjacent to the above sections chosen for HE staining were used for immunofluorescence analysis as the routine procedures. Sections were incubated with MVH antibody (ab13840, 1:200; Abcam, Cambridge, MA, USA) and ZP3 antibody (H‐300) (sc‐25802, 1:100; Santa Cruz) overnight at 4°C. The next day, the ovary sections were incubated with secondary antibodies (Alexa Fluo^®^594 donkey anti‐rabbit IgG (H + L), AntGene, ANT030, 1:200; Alexa Fluo^®^488 donkey anti‐rabbit IgG (H + L), AntGene, ANT024. 1:200) for 60 min. at 37°C. As the negative control, normal rabbit IgG was used here instead of primary antibody. Microscopy was performed using microscope (version 1.8.1, Olympus), and the images were acquired with the cellSens Dimension software. The immunofluorescence result was quantified *via* counting the follicle with MVH‐positive staining and ZP3‐positive staining.

### Morphological analysis and follicle counting

Primordial follicles and growing follicles are counted in this study. Identification of follicle types was categorized in accordance with the protocol of previous studies 31, 32. Growing follicles are follicles which have been activated. Morphologically, they are characterized by oocyte diameter greater than 20 μm and transition of the flat epithelial layer to cuboidal granulosa cells. While the primordial follicles are composed of small oocytes (< 20 μm) and flat granulosa cells, the numbers of follicles at each developmental stage were counted and averaged in two largest cross serial sections of the ovary centre with HE staining 26, 31. Meanwhile, to further identify the different stage follicles, the adjacent ovarian sections were labelled with ZP3 and MVH. For each ovary, we used the above two methods to determine its follicle number. Three individual persons counted the follicles in this study. The oocyte diameter was measured by ImageJ software program (a free software developed by contributors worldwide).

### Quantitative real‐time PCR

After the ovaries were cultured for 48 hrs, the blocking effect of Si‐*Sema6c*‐Chol was detected using quantitative real‐time PCR. While the rest ovaries were harvested after 4 days culture to detect the mRNA expression of *Gdf9* (growth differentiation factor 9), *Zp1* (zona pellucida glycoprotein 1), *Zp2* (zona pellucida glycoprotein 2) and *Zp3* (zona pellucida glycoprotein 3), primer sequences of *Sema6c* were designed by https://www.ncbi.nlm.nih.gov/tools/primer-blast/. Primer sequences of *Gapdh*,* Gdf9*,* Zp1*,* Zp2* and *Zp3* were obtained from Primer Bank. All the primer sequences of the above genes are listed in Table 1.

**Table 1:** The sequences of primers used for *quantitative real‐time* PCR

Genes	Primers	Sequence (5′to3′)
*Gapdh*	Forward Primer	TGGATTTGGACGCATTGGTC
	Reverse Primer	TTTGCACTGGTACGTGTTGAT
*Sema6c*	Forward Primer	TTCCAGGCCAGTGATGCTGTAG
	Reverse Primer	TGCTCCAACGCATAGACAAAGTG
*Gdf9*	Forward Primer	GTCACCTCTACAATACCGTCCG
	Reverse Primer	CACCCGGTCCAGGTTAAACA
*Zp1*	Forward Primer	CGGTTTGAGGTGAATAACTGCT
	Reverse Primer	GTGGCAGCCTTTGTAATCAGC
*Zp2*	Forward Primer	AAGCCTTCCTCAGTCCGAGAA
	Reverse Primer	CCAGAGCATAAGTGCAGTTCA
*Zp3*	Forward Primer	CAGATGACGAAAGATGCCCTG
	Reverse Primer	ACACGGTTAGTCCTGAGGATG

### Western blotting

To detect the effect of Si‐*Sema6c* on SEMA6C protein expression, the ovaries were harvested after 48 hrs culture. While the rest of ovaries of each group were lysed in RIPA buffer after 4 days culture for next Western blotting experiments as the routine procedure, membranes were incubated with different primary antibody overnight at 4 °C: anti‐SEMA6C (AF2108, 1:800, 0.25 μg/ml; R&D Systems), AKT (anti‐pan‐AKT antibody, Abcam, ab8805, 1:1000), p‐AKT (phospho‐S473, Abcam, ab81283, 1:1000), rpS6 (ribosomal protein S6) polyclonal antibody (A6058, 1:1000; ABclonal, Wuhan, China), phospho‐rpS6 (S235/236, AP0296, 1:1000; ABclonal) and visualized by Pierce^™^ ECL Western blotting substrate (No. 32209; Thermo Fisher Scientific, Waltham, MA, USA) or AP (alkaline phosphatase) substrate (1‐Step^™^ NBT/BCIP substrate solution, No. 34042: Thermo Fisher). Integrated light intensity of each band was quantified with Image Lab (Java image processing software, BioRad, Hercules, CA, USA) and used to compare treatment‐induced changes in the concentration of phosphoproteins. GAPDH expression was measured to verify equal loading.

### Statistical analysis

SPSS 18.0 software program (IBM, NY, USA) was used for statistical analysis and data comparison. To evaluate the statistical significance of differences between different groups, unpaired Student *t*‐tests were applied in our study. All the experiments were repeated three times or more. The average standard error of the mean (S.E.M.) is presented in the results. *P *<* *0.05 was defined as statistically significant.

## Results

### The expression of SEMA6C in the neonatal mouse ovaries

**Figure 1:**
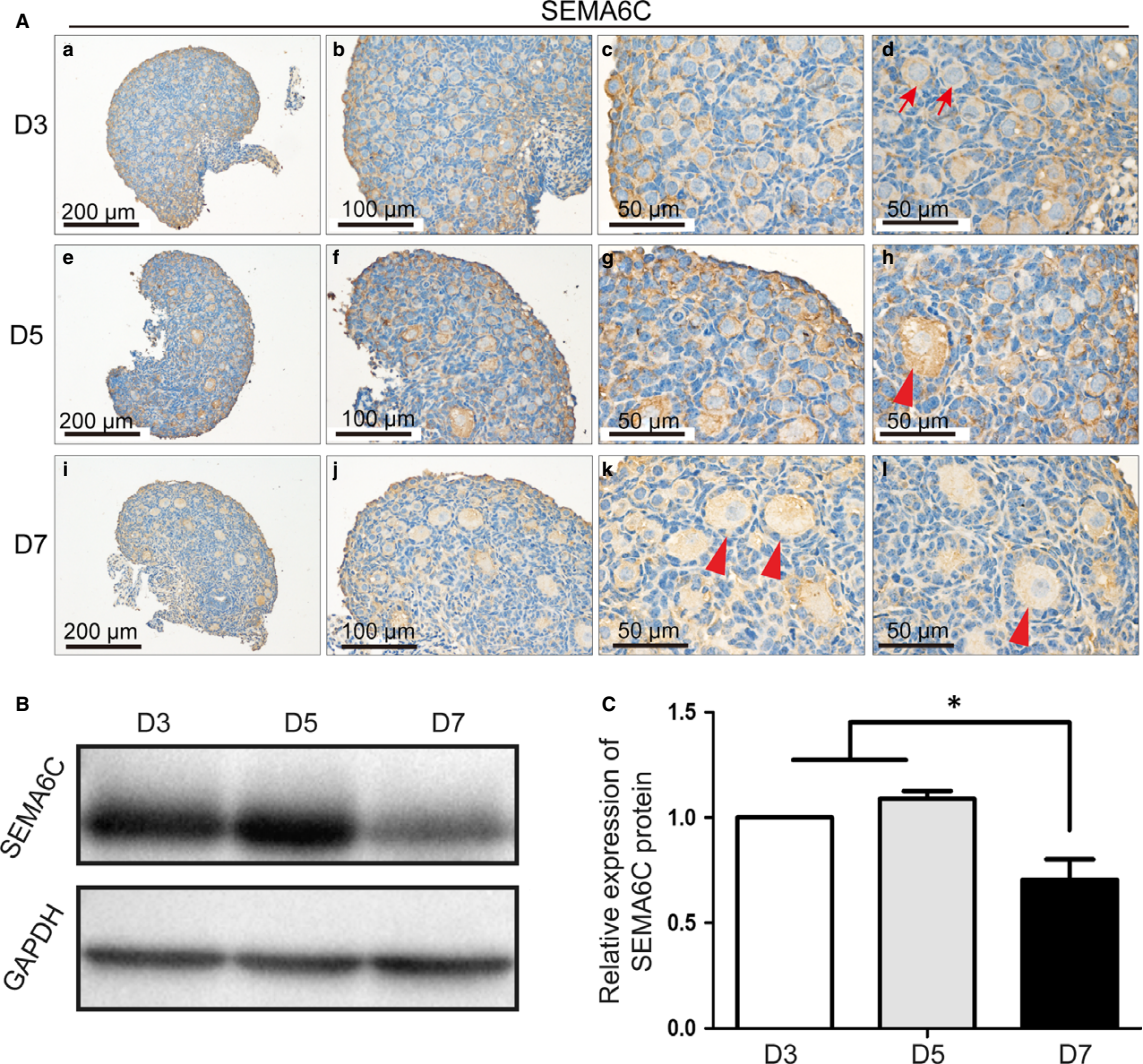
The expression of SEMA6C in the D3, D5 and D7 mouse ovaries. (**A)** SEMA6C protein expression was present in D3, D5 and D7 mouse ovaries, SEMA6C protein was expressed in primordial follicles (red arrows) and growing follicles (red arrow heads), mainly in the cytoplasm of oocytes, whereas it expressed relatively weaker in the interstitial cells and granulosa cells. (**B)** SEMA6C protein expression levels in D3, D5 and D7 ovaries. (**C**) The relative SEMA6C protein levels of D3, D5 and D7 ovaries were calculated from the band intensities (1.03 ± 0.05, 1.08 ± 0.06, 0.70 ± 0.17, respectively), and the SEMA6C expression of D7 ovaries decreased significantly compared with D3 or D5 ovaries (*P <* *0.05), whereas there was no significant difference of the SEMA6C expression between D 3 and D 5 ovaries. The data in the right panels represent the mean ± S.E.M. from three independent experiments.

To evaluate the SEMA6C protein expression in mouse ovaries, we first performed immunohistochemistry analyses with neonatal mouse ovaries. The ovaries of D3 (Day 3) female mice contained mainly primordial follicles (Fig. 1A_a‐d). In the ovaries of D5 (Day 5) mice, some primary follicles began to form (Fig. 1A_e‐h) and follicles further developed on Day 7, and more primary follicles and secondary follicles came into being (Fig. 1A_i‐l) as that we previously reported 26. The follicles were classified as follows: an oocyte surrounded by flattened pre‐granulosa cells was regarded as a primordial follicle; the growing follicle consists of an enlarged oocyte and one layer or more cuboidal granulosa cells. The immunohistochemistry results revealed that SEMA6C protein was mainly expressed in the cytoplasm of oocytes in the neonatal mouse ovary, whereas the granulosa cells and interstitial cells exhibited relatively weaker staining (Fig. 1A). However, in different primordial follicles, the intensity of SEMA6C protein expression was not the same, as the red arrows show two primordial follicles in Figure 1A_d. The Western blot results showed that SEMA6C expression decreased on Day 7 compared with Day 3, Day 5 (*P <* *0.05), whereas there was no significant difference in the SEMA6C expression between Day 3 and Day 5 ovaries (Fig. 1B‐C).

### The attenuation of SEMA6C promoted the primordial follicle activation

**Figure 2:**
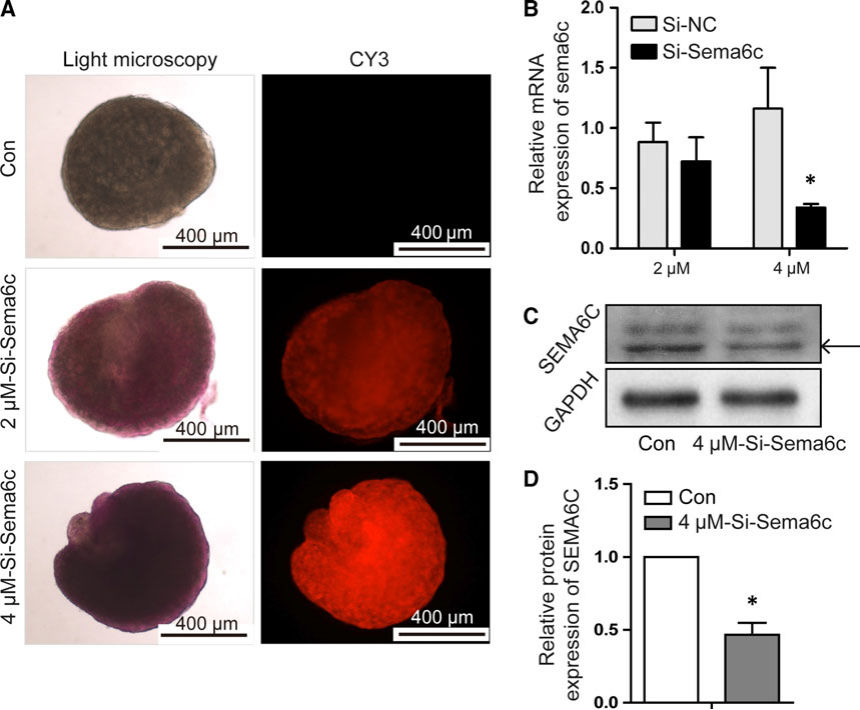
Determination of effective concentrations of the Si‐*Sema6c*‐Chol in cultured ovaries from 3‐day‐old mice. (**A**) CY3‐labelled‐Si‐*Sema6c*‐Chol diffused into the cultured ovaries upon addition to the medium. To down‐regulate the SEMA6C expression in neonatal mouse ovaries, we used CY3‐labelled‐Si‐*Sema6c*‐Chol in the *in vitro* culture system. Concentrations of 2 μM and 4 μM were tested in our experiments. Ovaries were collected after incubation for 48 hrs and evaluated by fluorescence microscopy. When at concentration of 2 μM and 4 μM, the strong red fluorescence (CY3 labelling) could be easily observed by fluorescence microscope and red fluorescence at the concentration of 4 μM was significantly stronger than that of 2 μM. (**B**) At concentration of 4 μM, the mRNA expression of *Sema6c* could be blocked 68.3 ± 4.2% compared with the control group after 48‐hrs culture (*P <* *0.05). (**C**) at concentration of 4 μM, the protein expression of SEMA6C could be well inhibited (*P <* *0.05) compared with the Con group after 48‐hrs culture. (**D**) The relative protein levels were calculated from the band intensities. The band intensity of Si‐*Sema6c* group was only 43.9 ± 12.7% of the Con group (*P <* *0.05). The data in the right panels represent the mean ± S.E.M. from three independent experiments.

Cholesterol‐conjugated SiRNA can be used to inhibit gene expression in the small tissue culture system, as the cell absorption of SiRNA is promoted and the half‐life of SiRNA is extended. Therefore, cholesterol‐conjugated Si‐*Sema6c* RNA (Si‐*Sema6c*‐Chol) was applied in this study to down‐regulate SEMA6C expression. To test whether the Si‐*Sema6c*‐Chol could well transfuse into the neonatal ovaries, we used CY3‐labelled‐Si‐*Sema6c*‐Chol in the culture system. After 48‐hrs culture, the strong red fluorescence (CY3 labelling) could be easily observed by fluorescence microscope and red fluorescence at the concentration of 4 μM was significantly stronger than that of 2 μM (Fig. 2A). And after 48 hrs culture, the *Sema6c* mRNA levels showed accordant result: the *Sema6c* mRNA expression at the concentration of 4 μM was better blocked (68.3 ± 4.2%) than that of 2 μM (18.2 ± 3.4%) (Fig. 2B). Meanwhile, the SEMA6C protein expression could be strongly inhibited, decreased more than 50% compared to the Con group (*P <* *0.05) (Fig. 2C,D), as the Western blotting results showed at the concentration of 4 μM. So, we used 4 μM Si‐*Sema6c*‐Chol to carry on the following experiments.

**Figure 3:**
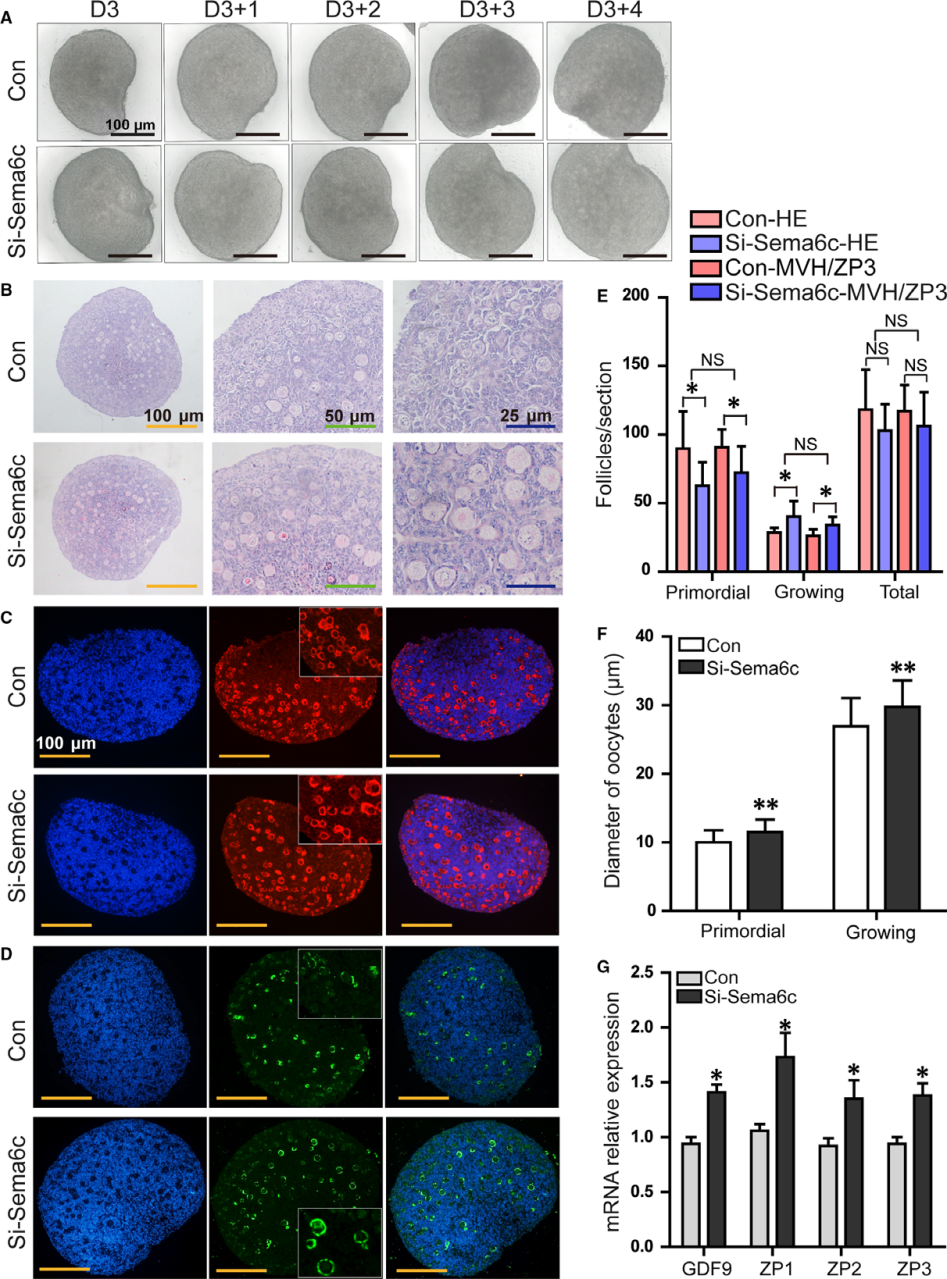
The attenuation of SEMA6C signalling activated early primordial follicle growth. (**A**) ovaries were cultured at the concentration of 4 μM Si‐*Sema6c*‐Chol; the morphology of the cultured ovaries was observed under a stereomicroscope during the 4 days of culture. After 4 days of culture, the ovaries in both groups appeared healthy under the stereomicroscope, without obviously dark internal areas. B‐D, HE staining (**B**) of ovaries cultured *in vitro* for 4 days. To identify oocytes and growing follicles more clearly, ovarian sections were immunofluorescence labelled with MVH (**C**) and oocyte‐derived ZP3 (**D**). (**E**) The numbers of primordial, growing and total follicles were determined according to HE staining and MVH/ZP3 labelling. In the Si‐*Sema6c* group, the number of primordial follicles was significantly reduced (*P <* *0.05), and the number of growing follicles increased compared with the control group (*P <* *0.05). No significant difference was observed in the total number of follicles (*P *>* *0.05), although a slight reduction was observed in the Si‐*Sema6c* group. (**F**) mean diameters of oocytes in Con and Si‐*Sema6c* groups. The mean oocyte diameters of primordial follicles were 10.03 ± 1.75 μm in Con group and 11.53 ± 1.81 μm in Si‐*Sema6c* group (**P <* *0.01); the mean oocyte diameters of growing follicles in the Si‐*Sema6c* group (29.78 ± 3.83 μm) were significantly greater compared with the Con group (26.95 ± 4.10 μm) (**P <* *0.01). (**G**) The relative mRNA expressions of *Gdf9*,* Zp1*,* Zp2* and *Zp3* in the Si‐*Sema6c* group were significantly higher than those of the Con group (*P <* *0.05). The data presented in panels (**E**‐**G**) correspond to mean ± S.E.M. from three independent experiments.

To investigate the function of *Seme6c* in early follicle development, 3‐day‐old neonatal mouse ovaries were cultured with 4 μM Si‐negative‐control (Si‐NC) or 4 μM Si‐*Sema6c*‐Chol for 4 days. During the 4 days *in vitro* culture, the morphology of cultured ovaries was observed and photographed under the microscope every day. The ovaries in all the groups appeared healthy, without dark regions (Fig. 3A). After the 4‐day culture, ovaries were harvested for paraffin sections. To determine the effect of low SEMA6C expression on the activation of primordial follicles, the above‐mentioned methods were used to count the follicles in different stages. Two largest cross section consecutive sections were stained with haematoxylin and eosin (Fig. 3B); meanwhile, the adjacent sections were separately labelled with MVH and ZP3 (Fig. 3C,D). The follicle counting results according to the above two methods are shown in Figure 3E. Compared with the Con group (HE: 89.73 ± 27.15/section; MVH/ZP3: 90.75 ± 13.01/section), the number of primordial follicles in the Si‐*Sema6c* group (HE: 62.79 ± 17.17/section; MVH/ZP3: 72.33 ± 19.10/section) was significantly reduced. However, the number of growing follicles was increased in the Si‐*Sema6c* group relative to the Con group (HE: 28.55 ± 3.59 (Con), 40.18 ± 11.41 (Si‐*Sema6c*); MVH/ZP3: 26.25 ± 4.65 (Con), 34.00 ± 6.15 (Si‐*Sema6c*), respectively) (*P <* *0.05). Although a little reduction in total follicle numbers was observed in the Si‐*Sema6c* group, no significant difference between the total follicle numbers was observed (HE: 118.27 ± 29.00, 102.96 ± 19.08; MVH/ZP3: 117.00 ± 19.00, 106.33 ± 24.58 respectively) (*P *>* *0.05) (Fig. 3E). The two different methods for follicle counting showed no significant difference (*P *>* *0.05). To evaluate the oocyte growth, we measured the oocyte diameters of different stage follicles by ImageJ in the above HE‐stained sections. The mean oocyte diameter was significantly greater in the Si‐*Sema6c* group than that in the Con group (primordial follicles: 11.53 ± 1.81 μm and 10.03 ± 1.75 μm, respectively (**P <* *0.01); growing follicles: 29.78 ± 3.83 μm and 26.95 ± 4.10 μm, respectively) (**P <* *0.01) (Fig. 3F). The mRNA expressions levels of *Gdf9*,* Zp1*,* Zp2* and *Zp3* genes, which were involved in primordial follicle activation and oocyte growth, were evaluated by quantitative real‐time PCR, and they were all up‐regulated in Si‐*Sema6c* group. The mRNA expressions of *Gdf9*,* Zp1*,* Zp2* and *Zp3* in Si‐*Sema6c* group were 1.50‐, 1.63‐, 1.46‐, 1.47‐fold of the Con group, respectively. (*P <* *0.05) (Fig. 3G).

### The molecular mechanisms underlying the accelerated primordial follicle activation in Si‐*Sema6c*‐treated ovaries

The initial recruitment of follicles was regulated by multiple signalling pathways, and PI3K‐AKT signal pathway has been proved to have interaction with semaphorin family members 16, 17, 33, 34. SEMA6C signal pathway has been demonstrated that it regulates the primordial follicle activation. Therefore, ovaries transfected with Si‐*Sema6c* were also treated with LY294002, the inhibitor of PI3K‐AKT pathway to investigate the molecular mechanisms regulating the accelerated primordial follicle activation with SEMA6C down‐regulated.

**Figure 4:**
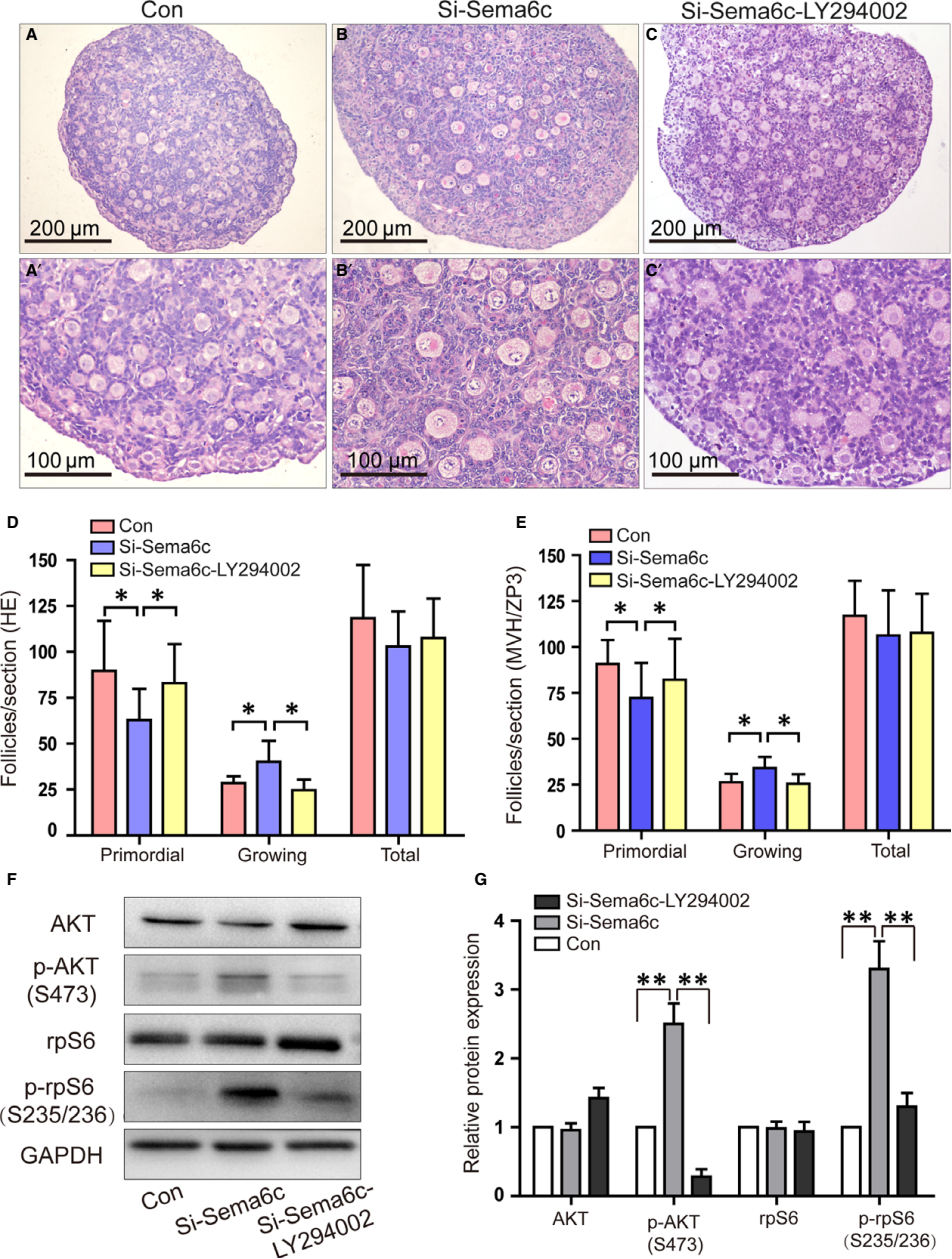
Effect of Si‐*Sema6c* on the PI3K‐AKT‐rpS6 signal pathway. (**A**‐**C)**, the morphology of ovaries treated by NC‐SiRNA, Si‐*Sema6c* or Si‐*Sema6c* with LY294002 after 4 days of culture. (**D**,**E**) The numbers of primordial follicles and growing follicles were determined according to HE staining and MVH/ZP3 labelling. In the Si‐*Sema6c* group, the number of primordial follicles decreased and the number of growing follicles significantly increased (*P <* *0.05). But when we added LY294002, the number of primordial follicles increased and the number of growing follicles significantly decreased compared with the Si‐*Sema6c* group (*P <* *0.05). The Si‐*Sema6c*‐LY294002 group showed similar follicle number with the Con group (*P *>* *0.05). The two methods (HE staining *versus* MVH/ZP3 labelling) showed no significant difference (*P *>* *0.05). (**F**) The levels of AKT, p‐AKT (S473), rpS6, p‐rpS6 (S235/236) were examined by Western blot analyses. (**G**) Quantification of Western blotting indicated an enhanced phosphorylation of AKT at Ser473 and rpS6 at Ser235/236 in Si‐*Sema6c* group compared with the Con group (**P <* *0.01). LY294002 reversed the increased phosphorylation of AKT and rpS6 caused by the Si‐*Sema6c* (**P <* *0.01). The data presented at panels (**D**,** E**,** G)** correspond to mean ± S.E.M. from three independent experiments.

LY294002 (20 μM) was added into the culture media of the ovaries after 48 hrs treated with Si‐*Sema6c*. The cultured ovaries were harvested for morphological and molecular analyses as above described after another 48 hrs. The ovary sections were stained with HE (haematoxylin–eosin) (Fig. 4A–C). The number of growing follicles of the Si‐*Sema6c*‐LY294002 group relatively decreased compared with that of the Si‐*Sema6c* group without LY294002 treated and showed similar morphology with the Con group (Fig. 4A–C). Then, we further evaluated the number of follicles in different stages using the above‐mentioned methods (HE staining and MVH/ZP3 labelling). Figure 4D shows the number of follicles counted according to HE staining. The number of primordial follicles in Si‐*Sema6c*‐LY294002‐treated ovaries (82.92 ± 21.32/section) was significantly increased compared with Si‐*Sema6c*‐treated ovaries (62.79.5 ± 17.17 (*P <* *0.05), while closed to the Con ovaries (89.73 ± 27.15) (*P *>* *0.05). The numbers of growing follicles in the three groups (Con, Si‐*Sema6c*, Si‐*Sema6c*‐LY294002) were 28.55 ± 3.58, 40.18 ± 11.41, 24.67 ± 5.82 respectively, and the growing follicle number of Si‐*Sema6c* group significantly increased compared with the other two groups (*P <* *0.05). Moreover, numbers of total follicles of the three groups were 118.27 ± 29.00, 102.96 ± 19.07, 107.58 ± 21.53 respectively (*P *>* *0.05) (Fig. 4D). The Figure 4E shows the number of follicles counted according to MVH/ZP3 labelling. The number of follicles in the Con, Si‐*Sema6c*, Si‐*Sema6c*‐LY294002 groups was as follows: primordial follicles (90.75 ± 13.01, 72.33 ± 19.10, 82.17 ± 22.26, respectively), growing follicles (26.25 ± 4.65, 34.00 ± 6.15, 25.58 ± 5.16, respectively) and total follicles (117.00 ± 19.00, 106.33 ± 24.58, 107.75 ± 21.28, respectively) (Fig. 4E). This follicle counting results showed no significant differences compared with the HE‐staining methods (*P *>* *0.05).

To further understand the mechanism of *Sema6c* effect on primordial follicle activation, Western blot analyses were carried out to detect the AKT and rpS6 protein expression levels and the phosphorylated forms of them. Figure 4F shows the levels of AKT, p‐AKT (S473), rpS6, p‐rpS6 (S235/236) by Western blot analyses. Quantification of Western blotting indicated an enhanced phosphorylation of AKT at Ser473 and rpS6 at Ser235/236 in Si‐*Sema6c* group compared with the Con group (2.5‐fold ± 0.3‐fold, 3.3‐fold ± 0.4‐fold, respectively) (**P <* *0.01, *n* = 3 independent blots) (Fig. 4G). LY294002 reversed the increased phosphorylation of AKT and rpS6 caused by the Si‐*Sema6c* (**P <* *0.01). Thus, SEMA6C suppression induced by Si‐*Sema6c* resulted in increased PI3K pathway activation, while LY294002 reversed the activation of PI3K pathway.

## Discussion

The initial recruitment of the primordial follicle is a gonadotropin‐independent process regulated by intrinsic ovarian growth factors 6. Dynamic homeostasis present between these inhibitory and stimulatory factors is quite important to guarantee the number of primordial follicles each wave. Recently, several studies have demonstrated that several key factors could affect the initial recruitment process through multiple signalling pathways 10, 35, 36, and the exact mechanism by which primordial follicles are activated and recruited into the growing pool is still less clear and need to be further explored.

The current study indicated that SEMA6C signalling was involved in the regulation of early primordial follicle dormancy/activation. Low expression of SEMA6C accelerated the primordial follicle activation process in the neonatal mouse ovary through PI3K‐AKT‐rpS6 signalling pathway.

The semaphorin family is a group of proteins that regulate axonal growth cone guidance and cell migration. These proteins could either promote or inhibit cell growth in different cases. Up to now, the researches of semaphorins in the female reproductive system are still relatively few. It is reported that SEMA4D and its receptor PLEXIN‐B1 participate in the mouse ovary follicular development 23. The gene expression profile of human cumulus–oocyte complex demonstrated that semaphorins (SEMA3A, SEMA6A and SEMA6D) significantly overexpressed in cumulus cells relative to oocytes 24. Semaphorin 3B, semaphorin 3F and semaphorin 4D are all demonstrated to be involved in ovarian cancer and associated with ovarian function 37, 38.

We found that the expression level of SEMA6C protein in ovaries sharply decreased between D5 and Day 7 after birth, which is the first time when primordial follicle activation begun. This result suggested the potential role of SEMA6C in the maintenance of primordial follicle quiescence and regulation of primordial follicle activation. Low expression of SEMA6C may accelerate the activation of primordial follicles.

SEMA6C expression of neonatal mouse ovaries was down‐regulated by SiRNA in the *in vitro* culture system to explore the role of SEMA6C in primordial follicle activation, because physiologically the primordial follicle activation was accompanied with the decreased expression of SEMA6C. We found that down‐regulation of SEMA6C of neonatal mouse ovaries resulted in a markedly decrease in the primordial follicles’ number and an increase in the growing follicles’ number and also promoted the oocyte growth. The expression levels of *Gdf9, Zp1, Zp2 and Zp3* were significantly increased in Si‐*Sema6c* treated ovaries, which demonstrated the overactivation of primordial follicles. All these results suggested that down‐regulation of SEMA6C accelerated primordial follicle activation and indicated that SEMA6C may act as a suppressor for primordial follicle activation. Down‐regulation of SEMA6C led to a slight reduction in the total follicle number; however, it was not statistically significant. We assume this slight reduction can be probably due to the inter ovary variability and can also be attributed to the death of primordial follicles. However, in which way SEMA6C affects primordial follicle survival needs further study.

Previous studies have shown that PTEN/PI3K/AKT signalling is involved in follicle survival and primordial follicle activation 10, 12, 39, 40. The phosphorylation of S6K1/rpS6 in oocytes is essential for the oocyte growth. PTEN could inhibit the phosphorylation of S6K1/rpS6 so as to keep the quiescence of primordial follicles 41. More studies demonstrated that enhanced phosphorylation of S6K1/rpS6 signalling by TSC/mTOR could promote the follicular activation 7, 42. Disorders of these signalling pathways can lead to accelerated primordial follicle activation and premature ovarian failure (POF). The above signal pathways play important roles in cell growth and proliferation. In the present study, we explored whether and how the *Sema6c* interacted with them.

Several studies have demonstrated that semaphorins participate in the regulation of PI3K signal pathway. Up‐regulation of SEMA3A leads to a marked decrease in AKT phosphorylation, indicating the PI3K activity was decreased 33. SEMA4D/PLEXIN‐B1 inactivates PI3K and dephosphorylates GSK‐3β and AKT through R‐Ras GAP activity 34. Semaphorins have been demonstrated to be able to regulate the PI3K/AKT pathway in the previous study 16, 17, 43. The effect of SEMA6C on endogenous AKT activity, a downstream target of PI3K, was examined in the ovary culture system. We found that SEMA6C suppression caused a marked increase in phosphorylation of AKT and rpS6. The S6K1‐rpS6, a downstream signalling of PTEN/PI3K/AKT and TSC/mTORC1, is also known to be involved in follicular dormancy and activation 41, 42. Treatment with LY294002 in Si‐*Sema6c*‐treated ovaries could reverse the accelerated primordial follicle activation, which indicated that SEMA6C may act on the upstream of PI3K and regulate the phosphorylation of AKT as well as the rpS6, but the exact mechanism that how SEMA6C interacts with PI3K/AKT/rpS6 pathway needs to be explored further. Our study is innovative to expound SEMA6C affects primordial follicle activation by regulating the PI3K/AKT/rpS6 pathway.

In summary, we found that low SEMA6C expression could promote primordial follicle activation in the neonatal mouse ovaries by activating the PI3K‐AKT‐rpS6 signalling pathway. We could speculate that relatively high expression of SEMA6C may help to keep the primordial follicle dormancy so as to control exhausting rate of primordial follicle pool and extend the reproductive lifespan in mammals. It should be noted that the limitation of this study is that we only examined the effect of low SEMA6C expression on primordial follicle activation. We did not investigate the effect of SEMA6C overexpression in mouse ovaries, as SEMA6C protein was strongly expressed in the 3‐day‐old mouse ovaries. More work will be carried out on this subject in further research.

## Conflicts of interest

The authors declare no conflict of interest.

## References

[1] Hsueh AJ , Kawamura K , Cheng Y , *et al* Intraovarian control of early folliculogenesis. Endocr Rev. 2015; 36: 1–24. 25202833 10.1210/er.2014-1020 PMC4309737

[2] Nelson SM , Telfer EE , Anderson RA . The ageing ovary and uterus: new biological insights. Hum Reprod Update. 2013; 19: 67–83. 23103636 10.1093/humupd/dms043 PMC3508627

[3] Sutherland JM , Keightley RA , Nixon B , *et al* Suppressor of cytokine signaling 4 (SOCS4): moderator of ovarian primordial follicle activation. J Cell Physiol. 2012; 227: 1188–98. 21604262 10.1002/jcp.22837

[4] Hsueh AJ . Fertility: the role of mTOR signaling and KIT ligand. Curr Biol. 2014; 24: R1040–2. 25517366 10.1016/j.cub.2014.09.033

[5] Fortune JE , Cushman RA , Wahl CM , *et al* The primordial to primary follicle transition. Mol Cell Endocrinol. 2000; 163: 53–60. 10963874 10.1016/s0303-7207(99)00240-3

[6] Adhikari D , Liu K . Molecular mechanisms underlying the activation of mammalian primordial follicles. Endocr Rev. 2009; 30: 438–64. 19589950 10.1210/er.2008-0048

[7] Adhikari D , Zheng W , Shen Y , *et al* Tsc/mTORC1 signaling in oocytes governs the quiescence and activation of primordial follicles. Hum Mol Genet. 2010; 19: 397–410. 19843540 10.1093/hmg/ddp483 PMC2798719

[8] Castrillon DH , Miao L , Kollipara R , *et al* Suppression of ovarian follicle activation in mice by the transcription factor Foxo3a. Science. 2003; 301: 215–8. 12855809 10.1126/science.1086336

[9] John GB , Shirley LJ , Gallardo TD , *et al* Specificity of the requirement for Foxo3 in primordial follicle activation. Reproduction. 2007; 133: 855–63. 17616716 10.1530/REP-06-0051 PMC2579775

[10] Reddy P , Liu L , Adhikari D , *et al* Oocyte‐specific deletion of Pten causes premature activation of the primordial follicle pool. Science. 2008; 319: 611–3. 18239123 10.1126/science.1152257

[11] McLaughlin M , Kinnell HL , Anderson RA , *et al* Inhibition of phosphatase and tensin homolog (PTEN) in human ovary *in vitro* results in increased activation of primordial follicles but compromises development of growing follicles. Mol Hum Reprod. 2014; 20: 736–44. 24830779 10.1093/molehr/gau037 PMC4106636

[12] John GB , Gallardo TD , Shirley LJ , *et al* Foxo3 is a PI3K‐dependent molecular switch controlling the initiation of oocyte growth. Dev Biol. 2008; 321: 197–204. 18601916 10.1016/j.ydbio.2008.06.017 PMC2548299

[13] Liu L , Rajareddy S , Reddy P , *et al* Infertility caused by retardation of follicular development in mice with oocyte‐specific expression of Foxo3a. Development. 2007; 134: 199–209. 17164425 10.1242/dev.02667

[14] Ren Y , Suzuki H , Jagarlamudi K , *et al* Lhx8 regulates primordial follicle activation and postnatal folliculogenesis. BMC Biol. 2015; 13: 39. 26076587 10.1186/s12915-015-0151-3 PMC4487509

[15] Jiang Z‐Z , Hu M‐W , Ma X‐S , *et al* LKB1 acts as a critical gatekeeper of ovarian primordial follicle pool. Oncotarget. 2016; 7: 5738–53. 26745759 10.18632/oncotarget.6792 PMC4868718

[16] Zhou Y , Gunput R‐AF , Pasterkamp RJ . Semaphorin signaling: progress made and promises ahead. Trends Biochem Sci. 2008; 33: 161–70. 18374575 10.1016/j.tibs.2008.01.006

[17] Roth L , Koncina E , Satkauskas S , *et al* The many faces of semaphorins: from development to pathology. Cell Mol Life Sci. 2009; 66: 649–66. 18953684 10.1007/s00018-008-8518-z PMC11131483

[18] Kruger RP , Aurandt J , Guan KL . Semaphorins command cells to move. Nat Rev Mol Cell Biol. 2005; 6: 789–800. 16314868 10.1038/nrm1740

[19] Giacobini P , Prevot V . Semaphorins in the development, homeostasis and disease of hormone systems. Semin Cell Dev Biol. 2013; 24: 190–8. 23219659 10.1016/j.semcdb.2012.11.005

[20] Uesaka N , Uchigashima M , Mikuni T , *et al* Retrograde semaphorin signaling regulates synapse elimination in the developing mouse brain. Science. 2014; 344: 1020–3. 24831527 10.1126/science.1252514

[21] Svensson A , Libelius R , Tagerud S . Semaphorin 6C expression in innervated and denervated skeletal muscle. J Mol Histol. 2008; 39: 5–13. 17605078 10.1007/s10735-007-9113-6

[22] Burgaya F , Fontana X , Martinez A , *et al* Semaphorin 6C leads to GSK‐3‐dependent growth cone collapse and redistributes after entorhino‐hippocampal axotomy. Mol Cell Neurosci. 2006; 33: 321–34. 17029982 10.1016/j.mcn.2006.08.008

[23] Regev A , Goldman S , Shalev E . Semaphorin‐4D (Sema‐4D), the Plexin‐B1 ligand, is involved in mouse ovary follicular development. Reprod Biol Endocrinol. 2007; 5: 12. 17376242 10.1186/1477-7827-5-12 PMC1838422

[24] Assou S , Anahory T , Pantesco V , *et al* The human cumulus–oocyte complex gene‐expression profile. Hum Reprod. 2006; 21: 1705–19. 16571642 10.1093/humrep/del065 PMC2377388

[25] Borgbo T , Povlsen BB , Andersen CY , *et al* Comparison of gene expression profiles in granulosa and cumulus cells after ovulation induction with either human chorionic gonadotropin or a gonadotropin‐releasing hormone agonist trigger. Fertil Steril. 2013; 100: 994–1001. 23856575 10.1016/j.fertnstert.2013.05.038

[26] Yang S , Wang S , Luo A , *et al* Expression patterns and regulatory functions of microRNAs during the initiation of primordial follicle development in the neonatal mouse ovary. Biol Reprod. 2013; 89: 126, doi: 10.1095/biolreprod.113.107730. 10.1095/biolreprod.113.107730 23986572

[27] Toyooka Y , Tsunekawa N , Takahashi Y , *et al* Expression and intracellular localization of mouse Vasa‐homologue protein during germ cell development. Mech Dev. 2000; 93: 139–49. 10781947 10.1016/s0925-4773(00)00283-5

[28] Castrillon DH , Quade BJ , Wang TY , *et al* The human VASA gene is specifically expressed in the germ cell lineage. Proc Natl Acad Sci USA. 2000; 97: 9585–90. 10920202 10.1073/pnas.160274797 PMC16908

[29] Song K , Ma W , Huang C , *et al* Expression Pattern of Mouse Vasa Homologue (MVH) in the Ovaries of C57BL/6 Female Mice. Med Sci Monit. 2016; 22: 2656–63. 27460133 10.12659/MSM.899830 PMC4973802

[30] El‐Mestrah M , Castle PE , Borossa G , *et al* Subcellular distribution of ZP1, ZP2, and ZP3 glycoproteins during folliculogenesis and demonstration of their topographical disposition within the zona matrix of mouse ovarian oocytes. Biol Reprod. 2002; 66: 866–76. 11906903 10.1095/biolreprod66.4.866

[31] Parrott JA , Skinner MK . Kit‐ligand/stem cell factor induces primordial follicle development and initiates folliculogenesis. Endocrinology. 1999; 140: 4262–71. 10465300 10.1210/endo.140.9.6994

[32] McGee EA , Hsueh AJ . Initial and cyclic recruitment of ovarian follicles. Endocr Rev. 2000; 21: 200–14. 10782364 10.1210/edrv.21.2.0394

[33] Chadborn NH , Ahmed AI , Holt MR , *et al* PTEN couples Sema3A signalling to growth cone collapse. J Cell Sci. 2006; 119: 951–7. 16495486 10.1242/jcs.02801

[34] Ito Y , Oinuma I , Katoh H , *et al* Sema4D/plexin‐B1 activates GSK‐3beta through R‐Ras GAP activity, inducing growth cone collapse. EMBO Rep. 2006; 7: 704–9. 16799460 10.1038/sj.embor.7400737 PMC1500830

[35] Zhang H , Liu K . Cellular and molecular regulation of the activation of mammalian primordial follicles: somatic cells initiate follicle activation in adulthood. Hum Reprod Update. 2015; 21: 779–86. 26231759 10.1093/humupd/dmv037

[36] Kerr JB , Myers M , Anderson RA . The dynamics of the primordial follicle reserve. Reproduction. 2013; 146: R205–15. 23929903 10.1530/REP-13-0181

[37] Joseph D , Ho SM , Syed V . Hormonal regulation and distinct functions of semaphorin‐3B and semaphorin‐3F in ovarian cancer. Mol Cancer Ther. 2010; 9: 499–509. 20124444 10.1158/1535-7163.MCT-09-0664 PMC2820590

[38] Dacquin R , Domenget C , Kumanogoh A , *et al* Control of bone resorption by semaphorin 4D is dependent on ovarian function. PLoS ONE. 2011; 6: e26627. 22046317 10.1371/journal.pone.0026627 PMC3202567

[39] Jagarlamudi K , Liu L , Adhikari D , *et al* Oocyte‐specific deletion of Pten in mice reveals a stage‐specific function of PTEN/PI3K signaling in oocytes in controlling follicular activation. PLoS ONE. 2009; 4: e6186. 19587782 10.1371/journal.pone.0006186 PMC2702689

[40] Kim SY , Ebbert K , Cordeiro MH , *et al* Cell autonomous phosphoinositide 3‐kinase activation in oocytes disrupts normal ovarian function through promoting survival and overgrowth of ovarian follicles. Endocrinology. 2015; 156: 1464–76. 25594701 10.1210/en.2014-1926 PMC4399322

[41] Reddy P , Adhikari D , Zheng W , *et al* PDK1 signaling in oocytes controls reproductive aging and lifespan by manipulating the survival of primordial follicles. Hum Mol Genet. 2009; 18: 2813–24. 19423553 10.1093/hmg/ddp217

[42] Adhikari D , Flohr G , Gorre N , *et al* Disruption of Tsc2 in oocytes leads to overactivation of the entire pool of primordial follicles. Mol Hum Reprod. 2009; 15: 765–70. 19843635 10.1093/molehr/gap092

[43] Bellon A , Luchino J , Haigh K , *et al* VEGFR2 (KDR/Flk1) signaling mediates axon growth in response to semaphorin 3E in the developing brain. Neuron. 2010; 66: 205–19. 20434998 10.1016/j.neuron.2010.04.006

